# Prospectus of the Illinois Medical and Surgical Journal

**Published:** 1844-04

**Authors:** 


### PROSPECTUS

OF THE

### ILLINOIS MEDICAL AND SURGICAL JOURNAL.

The Subscribers propose publishing, under the above title. a Medical Journal. in the city of Chicago, to be edited by JAMES V. Z. BLANEY, M. D., Pofessor of Chemistry and Materia Medica. in the Rush Medical College, assisted by his colleagues, and other Medical gentlemen. The objects of the Journal will be, to present to the profession, in a concise form, all improvements in the different branches of Medical Science, to make them acquainted with the most valuable of the new publications, and to form a medium of communication between its members, by furnishing them with a vehicle for giving to the public interesting cases and observations.

The utility of such a publication, if well conducted, cannot, we think, be called in question. There are numerous objects of local interest, such as the peculiarities of Western diseases, the effects of different remedies in their treatment, besides numerous interesting Medical and Surgical cases and operations occurring here, of great importance to the Profession, but which are but little noticed in Eastern Medical Journals.

There are higher interests of the Profession also to be guarded. Pretenders, who have "had their day" in the Eastern cities, are now coming in swarms into the Western States, destitute of truth as of professional character, they proclaim the grossest falsehoods, and use the names of eminent men in the profession for their purposes, thus deceiving the public and injuring the Profession. To refute these falsehoods, and place in the hands of Medical men facts, by which they can be exposed, will be one of the objects of this Journal.

Practitioners of Medicine who are settled in this and the adjoining States and Territories have many interests in common. Upon the character and standing of the Profession depend in a measure their own, they are assailed by the same enemies—quacks and deserters from the Profession. It is essential that they should be able to have the advantages of association, and to act in concert. This, their wide separation, and their devotion to the daily duties of the profession, prevents. It is hoped that this Journal, by making them known to each other, will form a bond of union, which will lead to more systematic association, and by diffusing information, contribute to the honor, well being, and improvement of the profession.

It will be issued Monthly, and as soon as a sufficient number of subscribers shall have been obtained. Each No. to contain Sixteen pages octavo, closely printed, with new type, and on fine paper, with a cover, at $1,00 per annum, payable on the delivery of the first number. Physicians who receive this, or other prospectuses, (which will be sent to them by mail,) are respectfully requested to act as agents, and transmit the names of such subscribers as they may procure, to the publishers, without delay.

*Chicago, March* 15, 1844.

ELLIS & FERGUS, Publishers,
Saloon Buildings, Clark Street, Chicago, Ill.

